# Anti-Inflammatory Effect of *Angelica gigas* via Heme Oxygenase (HO)-1 Expression

**DOI:** 10.3390/nu7064862

**Published:** 2015-06-15

**Authors:** Joon Hyeong Cho, Jung Eun Kwon, Youngmi Cho, Inhye Kim, Se Chan Kang

**Affiliations:** 1 Department of Biological and Environmental Science, Dongguk University, Goyang 410-820, Korea; E-Mail: jhcho@dongguk.edu; 2 Department of Biological Science, Gachon University, Seongnam 461-701, Korea; E-Mails: jjung@nmr.kr (J.E.K.); ym473@nmr.kr (Y.C.)

**Keywords:** *Angelica gigas*, decursin, coumarins, Anti-inflammation, HO-1 expression, vascular smooth muscle cells

## Abstract

*Angelica gigas* (AG) is effective against various medical conditions such as bacterial infection, inflammation, and cancer. It contains a number of coumarin compounds and the group of interest is the pyranocoumarin, which comprises decursin and decursinol angelate. This group has an effect on controlling inflammation, which is caused by excessive nitric oxide (NO) production. Heme oxygenases (HOs), particularly HO-1, play a role in regulating the production of NO. Thus, this study aimed to investigate the anti-inflammatory effects of AG by measuring HO-1 expression. Treatments with CH_2_Cl_2_ layer and *Angelica gigas* extract (AGE) showed the highest NO inhibition effects. Decursin, decursinol angelate, and nodakenin were isolated from the CH_2_Cl_2_ layer of AGE. Decursin also demonstrated the highest anti-oxidative effect among the coumarins. Although decursin had the best NO inhibition and anti-oxidative effects, the effects of AGE treatment far surpassed that of decursin. This is owing to the combination effect of the coumarins present within AGE, which is a solvent extract of AG. The expression of HO-1 is an effective indicator of the anti-inflammatory effects of AG. Based on the results of the coumarin compounds, HO-1 expression was found to be dose dependent and specific to decursin.

## 1. Introduction

*Angelica gigas* (Umbelliferae family) (AG) is an important medicinal plant, which has been traditionally used to treat circulatory disorders, anemia, and female afflictions because it has hematogenic potential [1,2]. This plant contains various compounds such as coumarins [3], essential oils [4], and polyacetylenes [5]. Among the coumarins, pyranocoumarins such as decursin and decursinol angelate have received considerable attention because of their pharmacological properties [1,3,6]. Decursin was first reported in *Angelica decursiva* and decursinol angelate, the isoform of decursin, was identified later in *Peucedanum terebinthaceum* [1,7]. Recently, various pharmacological properties of *A. gigas* have been reported, such as anti-bacterial [8,9], anti-cancer [10,11], anti-tumor [12], anti-oxidant, neuroprotective [13], anti-dementia [14], inhibition of platelet aggregation, and blood coagulation activity [15].

Inflammation is a beneficial host response in restoring tissues or defending against pathogenic infections [16,17,18]. Protection against pathogens is achieved through the immunological defense system by O_2_^−^ and nitric oxide (NO), which are produced by NADPH-oxide in macrophages and inducible nitric oxide synthase (iNOS) in the cytosol [16,17,19,20,21]. NO, produced from l-arginine by iNOS, has many biological functions. It is an important signaling molecule for the regulation of physiological mechanisms and is essential as a defense mechanism against a range of pathogens [22,23,24,25,26]. However, tight regulation is needed in the production of NO since excessive levels are cytotoxic, not only to pathogens but also to the cell itself owing to oxidative damage [16,17,27,28,29]. Inflammatory complications cause various chronic diseases and are closely related to carcinogenesis. It has been reported that the onset of stomach cancer can be increased by the inflammation of the stomach wall infected by *Helicobacter pylori* (HP) [30].

The production of NO can be regulated by heme oxygenases (HOs). HO, an enzyme that catalyzes the degradation of heme, has been found in higher plants, algae, bacteria, and mammals [31,32]. In mammalian tissue, three isoforms of HOs play an important role in maintaining cellular homeostasis and act as a defense mechanism against oxidative stress [29,31,33,34,35]. It has been reported that the expression of HO-1 can be induced by iNOS-derived NO in the mesangial and vascular smooth muscle cells (VSMCs) [33,36]; suppression of the activity of iNOS and inhibition of excessive NO production were indicative of anti-inflammatory properties [37].

Previous studies demonstrated that coumarins extracted from *A. gigas* suppressed the expressions of inflammatory mediators, such as matrix metalloproteinase (MMP)-9, iNOS, interleukin (IL)-1β, cyclooxygenase (COX)-2, and tumor necrosis factor (TNF)-α in RAW 264.7 cells, resulting in the inhibition of NO production [22,38]. However, its anti-inflammatory effects on vascular muscle cells, which are involved with chronic inflammation of blood vessels, have not been investigated.

In order to confirm the active components of *A. gigas*, we evaluated differences in NO production by treatments of each of the extracted fractions or compounds in RAW 264.7 cells. To determine whether *A. gigas* exerts anti-inflammatory effects on vascular cells, we also examined the levels of HO-1 expression by treatments of NO-inhibited active fractions on mouse vascular smooth muscle cells (MOVAs).

## 2. Experimental Section

### 2.1. Sample Preparation and Reagents

The plant material, AG (IT#288156, RDA Genebank, RDA, Jenju, Korea) was collected at a farm field located in Bongwha, Gyeongbuk province, South Korea, and proliferated at the experiment farm of the Agricultural Research Station (ARS), Dongguk University located in Goyang, Gyeonggi Province. The rhizomes of the plant were dried in a dark place for 10 days and then dried again at 40 to 50 °C in a dry oven for 3 days. Dried rhizomes (100 g) were ground into powder and used for the coumarin extraction.

The murine macrophage Raw 264.7 cell line and mouse vascular smooth muscle (MOVA) cell line were obtained from the American Type Culture Collection (ATCC; Rockville, MD, USA). The cells were cultured in Dulbecco’s Modified Eagle’s Media (DMEM, Gibco, Rockville, MD, USA) containing 1% penicillin/streptomycin (Gibco, Rockville, MD, USA) and 10% fetal bovine serum (Gibco, Rockville, MD, USA) at 37 °C and 5% CO_2_. They were sub-cultured every two to three days.

### 2.2. Determination of Nitric Oxide (NO) Production

Raw 264.7 cells (2 × 10^5^ cells/mL) were seeded in 96-well plates and pretreated with *Angelica gigas* extract (AGE), each solvent fraction, and the compounds for 2 h, and then stimulated with lipopolysaccharides (LPS) (1 μg/mL) for 24 h. Nitrite accumulation in the culture medium was measured as an indicator of NO production. NO production in each sample was assayed using the Griess reaction [39] with a Nitrate detection kit (Cayman, Ann Arbor, MI, USA), according to the provided instructions, and a standard curve using NaNO_2_ was generated in each experiment. Briefly, 100 μL of medium or standard NaNO_2_ was mixed with 100 μL of Griess reagent in a 96-well plate. After 15 min, the optical density (OD) was measured at 540 nm in a micro plate reader (model 550 microplate reader, Bio-Rad Laboratories, Hercules, CA, USA).

### 2.3. ORAC Assay

The Oxygen radical absorbance capacity (ORAC) assay was based on the method of Ou *et al.* (2001) [40]. The experiment was conducted in 75 mM of phosphate buffer (pH 7.4) at 37 °C. For sample extract lipophilic analysis, 20 μL of this solution was placed in each well in a 96-well plate. Fluorescein (FL) solution (200 μL) and 5 μL 63.4 mM 2,2’-azobis(2-amidinopropane) dihydrochloride (AAPH) (17.2 mg/mL and 9.4 μmol/well) were added in order to the well on the microplate reader, and readings were initiated immediately. The reader was programmed to record the fluorescence of FL every 2 min for 60 min at emission and excitation wavelengths of 535 nm and 485 nm, respectively. Trolox, a water-soluble analog of vitamin E, was used as a standard, and phosphate buffer was used as a blank. The results were expressed as μmole trolox equivalent/g, and at least three independent assays were performed for each sample.

### 2.4. Extraction and Isolation

**Figure 1:**
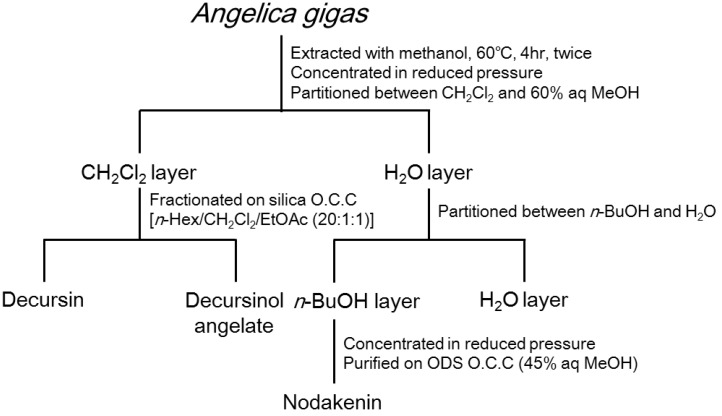
Isolation scheme for determining constituents of *A. gigas* with anti-inflammatory properties.

Dried and powdered rhizome of AG (200 g) was extracted with 1 L of 95% ethanol (EtOH extract, AGE) for 24 h at room temperature. Extracts were filtered through Whatman No. 1 filter paper and then concentrated under reduced pressure at 40 °C using a rotary evaporator and stored at 4 °C until use. This crude extract (AGE) was suspended in distilled water and sequentially partitioned with *n*-hexane, CH_2_Cl_2_, EtOAc, and *n*-BuOH. The CH_2_Cl_2_ layer was subjected to silica column chromatography (CC, *n*-hexane-CH_2_Cl_2_-EtOAc, 20:1:1) and then compounds **1** and **2** were separated by C18 reversed-phase HPLC (75% MeOH) and recycling HPLC (JAI-GEL, GS-310) with MeOH as an eluent. The *n*-BuOH layer was assessed using ODS column chromatography (CC, 45% aq MeOH) and then purified to yield nodakenin (**3**) (Figure 1). NMR experiments were performed on a Varian Unity INOVA 500 spectrometer with the usual pulse sequences. EIMS data were obtained on an HP 6890 series GC System equipped with a 5973 Mass Selective Detector. Column chromatography was carried out on Si gel 60 (Merck, 230–400 mesh) and Sephadex LH-20 (Lipophilic Sephadex, Sigma-Aldrich, 25–100 µm). HPLC and recycling HPLC were performed using a phenomenex Luna 10 µ C18 (2) (250 × 10 mm) column and JAIGEL-GS310 column, respectively, and a UV detector (254 nm).

**Decursin (1)**
  ^1^H-NMR (500 MHz, CDCl_3_) δ 7.57 (1H, d, *J* = 9.5 Hz, H-4), 7.15 (1H, s, H-5), 6.76 (1H, s, H-8), 6.20 (1H, d, *J* = 9.5 Hz, H-3), 5.64 (1H, s, C-2’’), 5.07 (1H, t, *J* = 4.9 Hz, H-3’), 3.18 (1H, dd, *J* = 17.0, 4.6 Hz, H-4’a), 2.85 (1H, dd, *J* = 17.0, 4.9 Hz, H-4’b), 2.13 (3H, s, 3’’-CH_3_), 1.86 (3H, s, H-4’’), 1.37 (3H, s, gem.-CH_3_), 1.35 (3H, s, gem.-CH_3_); ^13^C-NMR (125 MHz, CDCl_3_) δ 165.73 (C-1’’), 161.28 (C-2), 158.43 (C-3’’), 156.46 (C-7), 154.14 (C-9), 143.23 (C-4), 128.71 (C-5), 115.99 (C-6), 115.52 (C-2’’), 113.17 (C-3), 112.79 (C-10), 104.61 (C-8), 76.75 (C-2’), 69.11 (C-3’), 27.88 (C-4’), 27.46 (C-4’’), 25.00 (gem.-CH_3_), 23.13 (gem.-CH_3_), 20.32 (3’’-CH_3_)
**Decursinol angelate (2)**
  ^1^H-NMR (500 MHz, CDCl_3_) δ 7.59 (1H, d, *J* = 9.5 Hz, H-4), 7.16 (1H, s, H-5), 6.76 (1H, s, H-8), 6.20 (1H, d, *J* = 9.5 Hz, H-3), 6.10 (1H, d, *J* = 7.3 Hz, H-3’’), 5.12 (1H, t, *J* = 4.8 Hz, H-3’), 3.22 (1H, dd, *J* = 17.8, 4.8 Hz H-4’a), 2.90 (1H, dd, *J* = 17.8, 4.9 Hz, H-4’b), 1.87 (3H, d, *J* = 7.1 Hz, H-4’’), 1.82 (3H, s, 2’’-CH_3_), 1.39 (3H, s, gem.-CH_3_), 1.37 (3H, s, gem.-CH_3_); ^13^C-NMR (125 MHz, CDCl_3_) δ 167.01 (C-1’’), 161.26 (C-2), 156.45 (C-7), 154.17 (C-9), 143.21 (C-4), 139.40 (C-3’’), 128.67 (C-5), 127.29 (C-2’’), 115.88 (C-6), 113.22 (C-3), 112.79 (C-10), 104.55 (C-8), 76.67 (C-2’), 70.02 (C-3’), 27.87 (C-4’), 25.07 (gem.-CH_3_), 23.19 (gem.-CH_3_), 20.50 (2’’-CH_3_), 15.73 (C-4’’)
**Nodakenin (3)**
  ^1^H-NMR (500 MHz, DMSO-*d*_6_) δ 7.92 (1H, d, *J* = 9.7 Hz, H-4), 7.45 (1H, s, H-5), 6.77 (1H, s, H-8), 6.19 (1H, d, *J* = 9.7 Hz, H-3), 4.96 (1H, m, H-3’), 4.42 (1H, d, *J* = 7.8 Hz, H-1’’), 2.80~3.75 (9H, m, H-2’,3’,2’’,3’’,4’’,5’’,6’’), 1.30 (3H, s, gem.-CH_3_), 1.12 (3H, s, gem.-CH_3_); ^13^C-NMR (125 MHz, DMSO-*d*_6_) δ 163.51 (C-7), 161.00 (C-2), 154.17 (C-9), 145.17 (C-4), 126.09 (C-6), 124.41 (C-5), 112.70 (C-3), 111.72 (C-10), 97.65 (C-8), 97.25 (C-1’’), 90.23 (C-2’), 77.53 (C-5’’), 77.31 (C-3’’), 77.07 (C-4’), 73.94 (C-2’’), 70.68 (C-4’’), 61.64 (C-6’’), 29.60 (C-3’), 23.62 (gem.-CH_3_), 21.13 (gem.-CH_3_)

### 2.5. HO-1 Expression Assay

Western blotting was performed using standard techniques described by Cai *et al.* (2012) [41]. After treatment, MOVAs were harvested and lysed with ice-cold lysis buffer (50 mM Tris-HCl (pH 7.4), 150 mM NaCl, 1 mM EDTA, 0.1% Nonidet P-40, and 0.1% (w/v) sodium dodecyl sulfate (SDS)) containing a protease inhibitor cocktail (Roche Diagnostics Corp., Indianapolis, IN, USA) for 1 h. The lysates were then collected after centrifugation at 1500× *g* for 10 min at 4 sa. Tris-HCl (pH 7.5; 20 mM). Protease and phosphatase inhibitor mixtures (Sigma-Aldrich, St. Louis, MO, USA) were added. The protein concentration was determined using a protein assay kit (Bio-Rad Laboratories, Hercules, CA, USA) with bovine serum albumin (BSA) as the standard. Protein lysates (20 μg) were subjected to 10% SDS-polyacrylamide gel electrophoresis, transferred by electrophoretic means to an Immobilon-P-polyvinylidene fluoride membrane (Amersham, Arlington Heights, IL, USA) and probed with the primary antibodies (1:2000 dilution; Cell signaling, Danvers, MA, USA) and anti-HO-1 (1:1000 dilution; Cell signaling, Danvers, MA, USA). The blots were developed using an enhanced chemiluminescence (ECL) kit (Amersham Biosciences, Piscataway, NJ, USA). In all the western blotting experiments, the blots were re-probed with anti β-actin antibody as a control for protein loading.

## 3. Results

### 3.1. NO Inhibition Effects

Macrophage Raw 264.7 cells were treated with coumarins and the layers obtained from different solvents, and then NO production levels were compared (Table 1). NO production levels were the lowest in cells treated with the CH_2_Cl_2_ layer, while they were the highest in those treated with the *n*-hexane layer across all concentration treatments. NO levels in the cells treated with the CH_2_Cl_2_ layer were the lowest at 25 and 50 μg/mL with 18.5 ± 0.49 and 14.8 ± 0.54, respectively. When treated with 25 μg/mL AGE and 25 and 50 μg/mL *n*-hexane layer, the NO levels were highest with 24.0% ± 0.75%, 23.3% ± 0.28%, and 22.4% ± 0.46%, respectively. NO levels were not significantly different across all concentration of the AGE, EtOAc, and *n*-BuOH layer treatments. The NO level was the highest at low concentrations of AGE (25 μg/mL) with 24.0% ± 0.75%. However, when treated with high concentrations of the extracts (100 or 200 μg/mL), the levels significantly decreased (24.0% ± 0.75% and 9.5% ± 0.29%, respectively), similar to that observed in CH_2_Cl_2_ layer treatment group with 12.5% ± 0.87% and 8.95% ± 1.68%. Overall, NO levels tended to decrease across all extract treatments as the concentrations of the extracts increased. Therefore, inhibition of NO production is dose-dependent. Particularly in the case of the CH_2_Cl_2_ layer and AGE treatment groups, NO inhibition effects positively correlated to the increasing amount of extract, and the NO levels in the 200 μg/mL treatment groups decreased by more than 50% compared to the 25 μg/mL treatment group.

**Table 1:** The effect of *A. gigas* on the production of NO by Raw 264.7 cells in the presence of lipo-polysaccharide (LPS).

Treat-Ment Conc. (μg/mL)	Positive Control (DEX)	AGE	*n*-Hexane	CH_2_Cl_2_	EtOAc	*n*-BuOH	H_2_O	Decursin	Decursinol Angelate	Nodakenin
25	18.2 ± 0.36	24.0 ± 0.75	23.3 ± 0.28	18.5 ± 0.49	21.0 ± 0.39	21.3 ± 0.36	21.6 ± 0.50	19.4 ± 0.24	21.7 ± 0.21	22.1 ± 0.35
50	13.5 ± 0.22	20.7 ± 0.64	22.4 ± 0.46	14.8 ± 0.54	20.2 ± 0.14	20.1 ± 0.45	20.6 ± 0.53	17.1 ± 0.16	20.1 ± 0.30	23.1 ± 0.50
100	11.1 ± 0.21	12.9 ± 0.20	20.3 ± 0.53	12.5 ± 0.87	19.2 ± 0.20	18.3 ± 0.32	19.0 ± 0.38	15.6 ± 0.71	16.4 ± 0.33	21.3 ± 0.26
200	8.15 ± 0.17	9.5 ± 0 29	19.4 ± 0.49	8.95 ± 1.68	19.5 ± 1.27	14.0 ± 0.38	18.5 ± 0.39	10.9 ± 0.13	14.6 ± 0.85	21.6 ± 0.12

Cells were pre-treated with the crude extracts for 2 h, and then stimulated with LPS for 22 h for NO assay. Data are expressed as mean ± SD of three independent experiments performed in triplicate. AGE: *Angelica gigas* extract, DEX: Dexamethasone (10 μM).

Among the groups treated with coumarin compounds purified from each layer, the group treated with decursin showed the highest NO inhibition effect, followed by decursinol angelate, and nodakenin treatments. NO levels in the groups treated with decursinol angelate were similar to that of the group treated with *n*-BuOH layer. NO inhibition effects increased as the concentration of coumarins, decursin, and decursinol angelate increased, in a dose-dependent manner. Thus, high NO inhibition effects were observed in the AGE and CH_2_Cl_2_ layer treatment groups. High NO inhibition effect of AGE and CH_2_Cl_2_ layer appears to be owing to the interaction between decursin and decursinol angelate present in the solvent. Recently, anti-inflammatory effect of nodakenin in Raw 264.7 macrophage cell was reported in another experiment [42]. However, in this experiment, dose-dependent NO inhibition effect was not observed.

### 3.2. Anti-Oxidative Effect of AGE and Isolated Coumarins

Anti-oxidative effects of AGE and coumarin compounds are shown in Table 2. The oxygen radical absorbance capacities (ORAC) at low concentrations of AGE treatment (≤25 μg/mL) are 0.24 ± 0.022, 0.32 ± 0.023, and 0.46 ± 0.015, which were less than 50% of the anti-oxidative effect of vitamin E. However, in ≥50 μg/mL treatments, it increased to 0.91 ± 0.043 in a dose-dependent manner. Individual treatment of each coumarin compound—decursin, decursinol angelate, and nodakenin—showed lower anti-oxidative activities than that observed after AGE treatment. The anti-oxidative activities were similar across ≤50 μg/mL of each compound of treatment. However, at 100 μg/mL, decursin showed the highest level of anti-oxidative activity with 0.50 ± 0.008. Furthermore, although the anti-oxidative activities of each coumarin are different, it was proved that the effects of all three coumarins increase as dosage increases. Although decursin showed the highest anti-oxidative activity among the coumarins, AGE treatment showed significantly higher levels of antioxidant activity than those observed after individual treatments. These results seem to be due to the interaction of the three coumarins and it demonstrates the synergistic anti-oxidative effect.

**Table 2:** Antioxidant activity of *A. gigas* and their constituents.

Treatment Conc. (μg/mL)	Positive Control (Trolox, Vitamin E)	AGE	Decursin	Decursinol Angelate	Nodakenin
100	1.00 ± 0.020	0.91 ± 0.043	0.50 ± 0.008	0.40 ± 0.009	0.33 ± 0.015
50	1.00 ± 0.017	0.59 ± 0.026	0.27 ± 0.008	0.28 ± 0.009	0.22 ± 0.037
25	1.00 ± 0.027	0.46 ± 0.015	0.16 ± 0.010	0.18 ± 0.006	0.16 ± 0.009
12.5	1.00 ± 0.009	0.32 ± 0.023	0.10 ± 0.004	0.11 ± 0.006	0.08 ± 0.007
6.25	1.00 ± 0.022	0.24 ± 0.022	0.05 ± 0.001	0.07 ± 0.002	0.04 ± 0.007

Data are expressed as the mean of triplicate ± SD. The relative ORAC values are expressed as μmol Trolox/g sample. ANOVA was performed to compare values obtain between each dose for the same test.

### 3.3. HO-1 Expression of Isolated Coumarins

The effect on HO-1 expression in MOVAs by each individual coumarin—decursin, decursinol angelate, and nodakenin—purified from AG is shown in Figure 1. The control, β-actin gene, was constitutively expressed regardless of the treatment on CoPP, the activator of HO-1. However, in the case of HO-1, the expression of the gene was weak while strong HO-1 expression was observed when cells were treated with CoPP, which is known as the activator for HO-1 expression [41]. HO-1 expression, in particular, significantly increased after decursin treatment in the absence of CoPP activator and further, HO-1 expression was proved specific to decursin treatment owing to its dose-dependency. In the case of decursinol angelate and nodakenin, HO-1 expressions significantly increased after treatment with these two coumarins, but the gene expressions were not dose-dependent. Based on the results, the coumarin compounds—decursin, decursinol angelate, and nodakenin—purified from AG seem to be relevant to anti-inflammatory activity since they activated HO-1 gene expression in MOVAs. However, only HO-1 expression proved to be specific to decursin, while it is non-specific to the other two coumarins—decursinol angelate and nodakenin—in MOVAs.

To further understand how decursin affects HO-1 expression, the level of HO-1 protein was measured with/without 1 μM ZnPP, a HO-1 inhibitor, and 1 μg/mL LPS. LPS-induced inflammation led to the increased HO-1 expression compared to non-treated control. However, 25 μg/mL decursin stimulated HO-1 expression regardless of LPS treatment, indicating that decursin activates HO-1 expression. Even though ZnPP suppressed the level of HO-1, MOVAs treated with decursin and ZnPP showed increased level of HO-1 expression compared to ZnPP treated cells.

**Figure 2:**
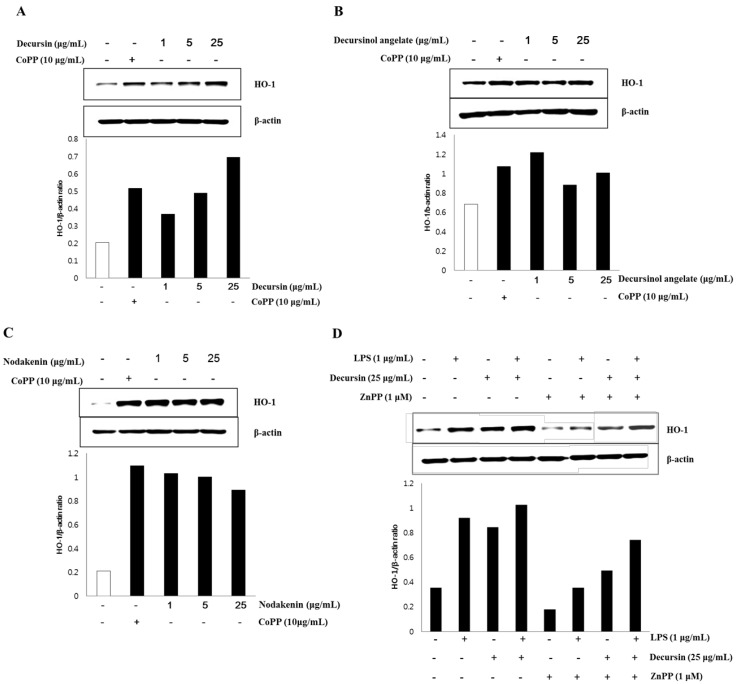
Effect of decursin, decursinol angelate, and nodakenin on HO-1 expression.

## 4. Discussions

Decursin and decursinol angelate, components of coumarin found in *A. gigas*, have various medicinal effects such as anti-bacterial, anti-inflammatory, and anti-cancer activities. Thus, in this study, coumarin compounds such as decursin, decursinol angelate, and nodakenin were successfully isolated from *A. gigas* and their anti-inflammatory effects were examined through HO-1 activation on the MOVAs. HO-1 activation is mediated by various mechanisms, including the blockade of immune response and increased production of carbon oxide [43]. HO-1 protects VSMCs against oxidative damage and proliferation [44,45].

While inflammation induced by NO is a beneficial defense mechanism against pathogens, it is also one of the main causes of several diseases including chronic diseases and cancer. Inflammation is caused by and is related to the production of NO and the expression of MMP-9, iNOS, IL-1β, as well as TNF-α [11,33]. Particularly, NO, which is produced from l-arginine by iNOS can cause inflammation by oxidative damage. This can be caused by either excessive NO production or through the combined activity of NO and superoxide [16,17,28]. Elevated inflammation activity leads to chronic diseases in patients with diabetes, which can develop into more severe cardiometabolic and interrelated complications or even cause cancer [30,46].

The measurement of NO production and ORAC assay are important means for predicting the anti-inflammatory effects of the fractioned extracts and coumarin compounds extracted from *A. gigas*. When macrophage Raw 264.7 cells were treated with the extracts and the coumarins, the CH_2_Cl_2_ layer, AGE, and decursin demonstrated the highest NO inhibition effects compared to the other treatments. In addition, NO inhibition effects increased as the concentration of each layer and coumarin increased in a dose-dependent manner, except in the case of nodakenin (Table 1). Although in the ORAC assay, the highest anti-oxidative effect among coumarins was displayed by decursin, but AGE was the most effective compared to each coumarin treatment (Table 2). Higher NO inhibition effects of both AGE and the CH_2_Cl_2_ layer, and higher anti-oxidative effects of AGE, appears to be due to the interaction between the coumarin compounds—decursin and decursinol angelate—contained in the solvent.

In order to identify the anti-inflammatory effect of the coumarin compounds, HO-1 expression was induced. In the absence of CoPP, an HO-1 expression activator [41], HO-1 expression significantly increased following the treatment with each coumarin (Figure 2). Thus, each coumarin has anti-inflammatory effect. Furthermore, based on the dose-dependent increase of HO-1 expression in the group treated with decursin only, HO-1 expression proves to be specific to decursin treatment, while it is non-specific to the other two coumarins: decursinol angelate and nodakenin. It has been known that HO, an enzyme that catalyzes heme degradation, has a defense mechanism against oxidative stress and can regulate NO-induced inflammation [29,35,46]. Bilirubin, the product of heme catabolism in mammals, in particular, may act as a physiological antioxidant [31,35]. It has been reported that NO, whose synthesis is induced by IL-1β, TNF-α, and iNOS, significantly increases the expression of HO-1 in mesangial cells and VSMC three- to six-fold [33,36]. The increased expression of HO-1 may be a response mechanism to control oxidative inflammation by inhibiting NO.

Inhibitory effects on NO production (Table 1) and anti-oxidative effects (Table 2) when treated with coumarin compounds, especially in the case of decursin, were demonstrated. However, cytotoxic effects were not observed in the MTT assay (data not shown). MTT assay is a colorimetric assay for assessing cell viability by using the mitochondrial metabolic ability of cells in reducing the yellow-colored tetrazolium dye MTT (3-(4,5-dimethyl thiazol-2-yl)-2,5-diphenyl tetrazolium bromide) to formazan [47]. However, since the results may differ based on different conditions or chemical treatments, HO-1 expression assay can be more accurate in evaluating anti-inflammatory effects related to NO production.

Decursin and decursinol angelate from *A. gigas* inhibit not only NO production and NO-induced inflammation by suppressing MMP-9, iNOS, IL-1β, and TNF-α expression but they also demonstrate anti-cancer effects by inhibiting cell proliferation and activating apoptosis [10,11,22,38]. Although many studies of coumarin compounds related to NO inhibition, HOs expressions, and their defense mechanism against pathogens have been published, research on their anti-inflammatory effect in relation to HO-1 expression were limited.

*A. gigas*, known as “Dang gui” in South Korea, is a plant belonging to the Umbelliferae family and is frequently used in oriental medicine. However, *A. gigas* from Korea, *A. sinensis* from China, and *A. acutiloba* from Japan are also classified as Dang gui depending on the criterion in the country. In this study, decursin and decursinol angelate appears to be most prevalent in *A. gigas*. Therefore, in order to develop functional foods or medicine, tests identifying the anti-inflammatory effect of coumarins from *A. gigas* using assays that test for the expression of HO-1 need to be conducted. To understand the mechanism by which *A. gigas* modulates HO-1 expression, future research should study how *A. gigas* is related to NF-E2 related factor 2 (Nrf2), activator protein-1 (AP-1), and signal transducer and activator of transcription (STATs), which are known regulators of HO-1. This work should determine if the extract of *A. gigas* ameliorates inflammation of blood vessels in *in vivo* models.

## 5. Conclusions

Three coumarin compounds—decursin, decursinol angelate, and nodakenin—were isolated from *A. gigas*, which is known as “Korean Dang gui”. CH_2_Cl_2_ layer and AGE produced the highest NO inhibition effect among the different solvent extracts; among the coumarin compounds, decursin exhibited the highest NO inhibition effect. As expected, the anti-oxidative effects of decursin were similarly highest among the coumarins. The anti-inflammatory effects of the coumarin compounds were confirmed by examining their effects on HO-1 expression in the MOVAs. Taken together, HO-1 expression is dose-dependent and specific to decursin. The expression of HO-1 is an effective indicator for the anti-inflammatory effects of *A. gigas* on MOVAs.
